# Who benefits most from Lyon’s bike sharing system?

**DOI:** 10.1371/journal.pone.0231550

**Published:** 2020-04-30

**Authors:** Jordan Cambe, Patrice Abry, Julien Barnier, Pierre Borgnat, Marie Vogel, Pablo Jensen

**Affiliations:** 1 ENS de Lyon, CNRS, Laboratoire de Physique, UCB Lyon 1, Univ Lyon, Lyon, France; 2 IXXI, Institut Rhônalpin des Systemes Complexes, Lyon, France; 3 ENS de Lyon, UJM Saint-Etienne, Centre Max Weber, ENS Lyon, Univ. Lumière Lyon 2, Univ Lyon, Lyon, France; Bruno Kessler Foundation, ITALY

## Abstract

Bike sharing systems (BSS) have been growing fast all over the world, along with the number of articles analyzing such systems. However the lack of databases at the individual level and covering several years has limited the analysis of BSS users’ behavior in the long term. This article gives a first detailed description of the temporal evolution of individual customers. Using a 5-year dataset covering 120,827 distinct year-long subscribers, we show the heterogeneous individual trajectories masked by the overall system stability. Users follow two main trajectories: about half remain in the system for at most one year, showing a low median activity (47 trips); the remaining half corresponds to more active users (median activity of 91 trips in their first year) that remain continuously active for several years (mean time = 2.9 years). We show that users from urban cores, middle-aged and male are over represented among these long-term users, which profit most from the BSS. This provides further support for the view that BSS mostly benefit the already privileged.

## 1 Introduction

Bike Sharing Systems (BSS) have been developing rapidly all over the world in the last decades, being now present in more than 500 cities. The number of studies of BSS has followed a similar pattern [1], focusing on several topics: evaluating BSS environment and public health impacts, understanding BSS traffic characteristics such as rebalancing, optimization of stations’ spatial distribution …The automatic recording of BSS activities has allowed a quantitative description of many BSS characteristics: Circadian and monthly activity patterns [2, 3], average speed [4], number of trips per day per bike [5], patterns of bicycle flows over the cities [2–4, 6] and influence of weather conditions [2]. The knowledge derived from these studies, especially on bicycle flows between stations [3, 7] and the prediction of bike reallocation schedules [8], can help the management of station balancing [3, 9–11], one of the main financial challenges of BSS [12]. Socio-demographics profiles of BSS users generally differ from the overall cities demographics. Studies carried out in Europe and North America [13–19] have shown that users are more likely to be young, male, with a high level of education and living in the city center. Finally, several studies have described the impact of BSS policies on environment and public health [20]. Other authors [21, 22] have listed the benefits of BSS: Emission reductions, individual financial savings, physical activity benefits, reduced congestion and facilitation of multimodal transport connections. However, some of these positive impacts of BSS have been questioned by [19]. For example, [16] showed the relatively low impact on people favorite mode of transportation. In particular [14, 23–25] showed, for several cities in Europe and Canada the low substitution rates from car usage to BSS. Most BSS riders are indeed people who used to walk or take public transportation.

Among all the above research axes, questions remain open on the commitment of BSS subscribers in the long term: Who are the long-term users, those that benefit most from the system? This question could not be answered by lack of accurate trip datasets over long periods of time, as mentioned in [23, 26]. Some articles have tried to characterize travel behaviors using surveys, such as [18, 27]. But the loyalty of users to BSS systems, which affects their long-term sustainability, has never been deeply investigated. This is the topic we address in detail in this article: How long do users remain active over the years? Does their activity increase, decrease or remain stable? Is it possible to predict these evolutions? These questions are addressed using a unique five years long dataset covering 120,827 distinct year-long users, among which 15,466 have stayed in the system for the whole period.

This article follows previous work [28] on Lyon’s BSS, *Vélo’v*, which, using a single year dataset (2011), characterized users according to their intensity and frequency of uses at different time scales (day, week, month and year). This work found 9 classes of users, ranging from ‘extreme users’, that use Vélo’v twice a day on average to ‘sunday cyclists’, who only use the system a few week-ends per year. Using a single year dataset to classify users has however two main limitations. Firstly, there is no way to distinguish between two possible interpretations for a user that appears to be very active from September to December. This could correspond either to (a) someone arriving in town in September that remains very active for the months/years to come or (b) someone who for an unknown reason uses the system only in those months. The second limitation arises from the impossibility to test the stability of users’ characteristics over years, which would allow to interpret them as real user properties. For example, do users classified in 2011 as ‘sunday cyclists’ retain this characteristic over the years? Have they only used Vélo’v in this way in 2011 or is this pattern a more personal—and stable—use of the system that lasts for longer periods? After presenting our dataset in next Section, we study in detail the evolutions of users’ behaviors over the years and then investigate the main limitations of single year datasets [28].

## 2 Dataset

The Vélo’v program started in 2005 in Lyon, France. The Vélo’v network now has 340 stations, where roughly 4000 bicycles are available. The stations are in the street and can be accessed at anytime (24/7) for rental or return. More information about the history of Vélo’v and the deployment of stations can be found in [2]. The Vélo’v autonomous system is deployed mostly in Lyon (∼ 500,000 inhabitants) and Villeurbanne (∼ 150,000 inhabitants) and completes a quote dense system of public transportation (including subways, tramways, trolleys and buses). Still, the system had an increasing popularity, as seen through the large increase of the number of use during the early years of deployment (as analysed in [2]). It has now a slightly increase of the yearly number of trips, as it will be seen in Table 1. The urban area of Lyon-Villeurbanne, being the second largest in France, is economically quite active and attracts a lot of universities (∼ 130000 students). The interaction between Bicycle Sharing Systems and public transportation system is difficult to study as this demands specific surveys to study inter-modality (see [18]). In a previous study [6], we were able to relay on spatial information about the trips, as the stations of departure and arrival were known. It then has been shown that groups of typical patterns of displacements for Lyon are: an intensive usage for commuting (including to university campuses) and connecting to railway stations or subway hubs; finally, on week-ends, leisurely trips along the Rhone and Saone rivers connecting the major parks. In these studies, we did not have any information about users (the unity of study was a trip). Joint information about users and space is not available for anonymity reasons.

**Table 1:** Number of trips per active user.

Year	2011	2012	2013	2014
Active Users	50,393	55,909	61,811	70,056
Trips	4,702,498	5,138,931	5,576,733	6,624,847
**Trips per user**	**93.3**	**91.9**	**90.2**	**94.5**
**Median trips per user**	**45**	**46**	**45**	**49**
**Standard Deviation**	**125.6**	**122.8**	**120.7**	**123.8**

The new dataset used in this work records all bicycle trips from 2011/01 to 2015/12 for the Vélo’v system, from now the point of view of users, as in [28]. The data were anonymized by their provider before communicating them to us. No location data about the bicycle trajectories were given. This analysis only relies on time stamps of bicycle trajectories from anonymized users. We are not aware of any explicit consent from the users to have their data analysed for research purposes. No ethics committee or data protection agency were consulted before carrying out this research. The dataset contains more than 38 million trips made by more than 3.8 million users. Each trip is documented with starting and ending times, duration, a user ID code and a tag describing the class of user (year-long subscriber, weekly or daily subscription, maintenance operation, etc). We also know user age, gender and residence zip code (corresponding mainly to the 10 different residence areas in Lyon-Villeurbanne). Note that for anonymity reasons, there are is no spatial information about the trip (start/end stations for example). Data are filtered according to the process used in [28], keeping only holders of year-long subscription cards (such as Tecely and Velo’v cards) and eliminating any anomalies. This leads to a subset of the original population, containing 120,827 users having done more than 27 million trips over 5 years. For each person, we count years from the first active day: For example, a user appearing in the records for the first time on March 14^th^, 2011 will end the first adapted year on March 13^th^, 2012. To avoid boundary artifacts for users that are active over several years, we stop recording trips at the anniversary date in 2015, even if there are recorded trips later in 2015.

## 3 Overall evolution

We first analyze the global system evolution over the 4 years. Table 1 shows that there is a steady increase in the number of users and trips. However, the average number of trips per user remains remarkably stable around 92 trips/year, despite the large variability (standard deviation larger that the average). A similar general temporal trend is found in [26].

## 4 Individual evolutions

**Fig 1:**
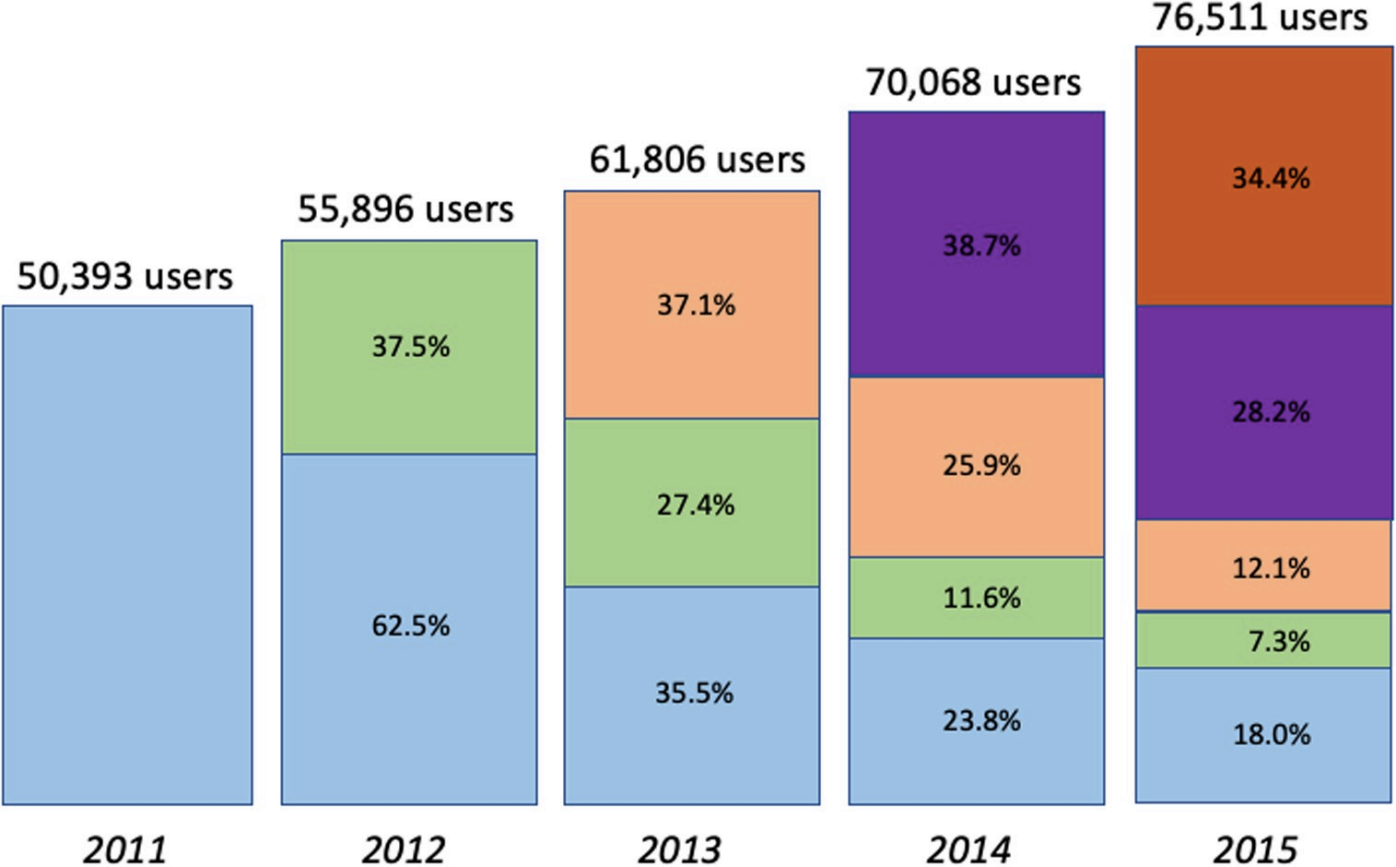
Progressive renewal of users over the years. For each year, the box height represents the total number of users and the colors the year users have entered the system. For example, in 2015, 34.4% of users are new to the system, while 18% started in 2011.

The overall system stationarity (slow increase of user numbers) hides a great variability at the individual level that can be uncovered only using long-term datasets at the individual level as ours. Every year, there is a strong user renewal, as the majority of users leave the system after their first year of a activity and are replaced by a greater number of new users. Fig 1 shows that every year the new users represent around 35% of the total. Then, they progressively leave the system, in a quite predictable way: They represent 26-28% of users the year after and 11-12% two years later. The only exception is the 2011 cohort, which by lack of data over the previous years, also includes users that entered the system *before* 2011 and may be more loyal than average.

### 4.1 Most users leave the system after one year

Analyzing user activity over calendar years as in Fig 1 is confusing, since users enter the system at any time during the year. To follow *individual* evolutions, we have to shift the different starting dates to a common origin using ‘adapted’ years as explained above.

**Fig 2:**
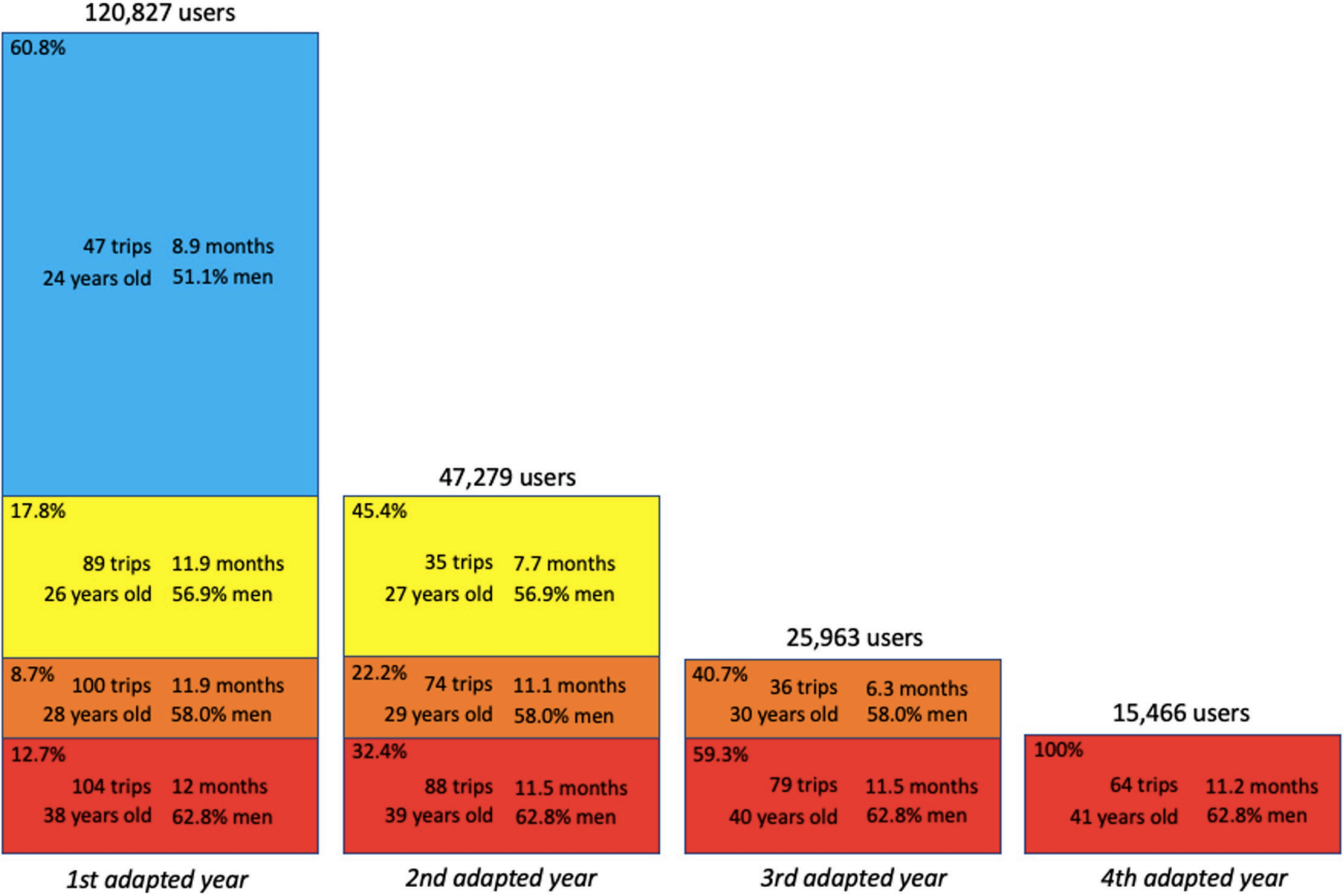
Percentages of users leaving the system at the end of different adapted years. For each group of users, we give the median number of trips per year, the median number of active months, the median age and the percentage of men. For example, ‘blue users’, which stopped at the end of their first adapted year, represented 60.8% of first year users, 51.1% were men, and were characterized by the following median numbers: 8.9 months of activity, 47 trips, 24 years old. Yellow users stopped at the end of their second adapted year. They had a median number of trips during their first year of 89 and during their second year of 35. They stopped after 7.7 active months during their second year. The figures for other users can be read from the figure.

Fig 2 shows that a large majority of users (60.8%, blue rectangle) quit after a single year of practice. These users are significantly younger than users staying at least two years (yellow, orange and red rectangles) (24 years old against 31), more likely women (51.1% of men compared to 59.1%) and less active: their median number of trips is 47, to be compared to 91. This low activity is mainly explained by a shorter time span of their activity (median close to 9 months instead of the whole year). This means that many of them stop using the system before the 12-month validity of their subscription, because they leave Lyon, buy a bike, change job…

Almost 20% of users stay in the system for 2 years (yellow rectangles in Fig 2). Note that their activity is significantly lower than that of more loyal users, that will stay in the system for 3 or 4 years (89 trips against 100, p-value <2.2*10^−16^). In this case, this reduced activity cannot be explained by a shorter activity time span. These users are consistently less active over the whole year, a feature that allows to predict a higher probability of quitting the system the following year, as we will check below. When these users reach their second (and last) active year, their activity becomes quite similar to the blue users, as their time span is reduced to 7.7 months and their activity much lower than in their first year (35 trips instead of 89).

Almost 9% of users stay in the system for 3 years (orange rectangles in Fig 2). Again, their activity, even two years before leaving the system, is significantly lower than that of more loyal users (100 trips against 104, p-value <2.2*10^−16^). This activity progressively diminishes over the years, reaching a very low value on the third and final year (36 trips over 6.3 months).

Finally, 12.7% of users stay in the system for at least 4 years (red rectangles in Fig 2). Their activity is consistently higher than average, and these users are older and more often men. Their activity also progressively diminishes over the years, a feature that we study in more detail below.

The most striking result is the high proportion (60.8%) of users that quit after a single year of practice (called ‘leavers’ hereafter). To the best of our knowledge, this surprising figure was previously unknown. It is worth noting however that this figure might be slightly overestimated. The reason is that users are identified through the ID of different cards, the most common being Velo’v own card (30.3% of the users), public transportation card (Tecely, 59.7%) and train card (Oura, 5.2%). The point is that the Tecely cards have to be renewed every 5 years. In some (uncontrolled) cases, this leads to a change of ID, which our analysis interprets as if the user had left the system and another had entered it. To estimate the proportion of incorrectly labeled exits from the system, we note that only 46.6% of Velo’v cards users give up after one year, the corresponding figure being 61.3% for Tecely users. As Velo’v cards do not go through the renewal process, this percentage could represent a lower bound on the ‘leavers’ proportion, if we assume that the proportion of leavers does not depend on the card used. To obtain another estimation, we may assume that all renewed Tecely cards (20% per year) change their ID. This would mean that the 61.3% figure is an overestimation of the real figure (61.3–20)/0.8 = 51.6%. These estimates converge to a proportion of leavers of 49%±2.5%.

**Fig 3:**
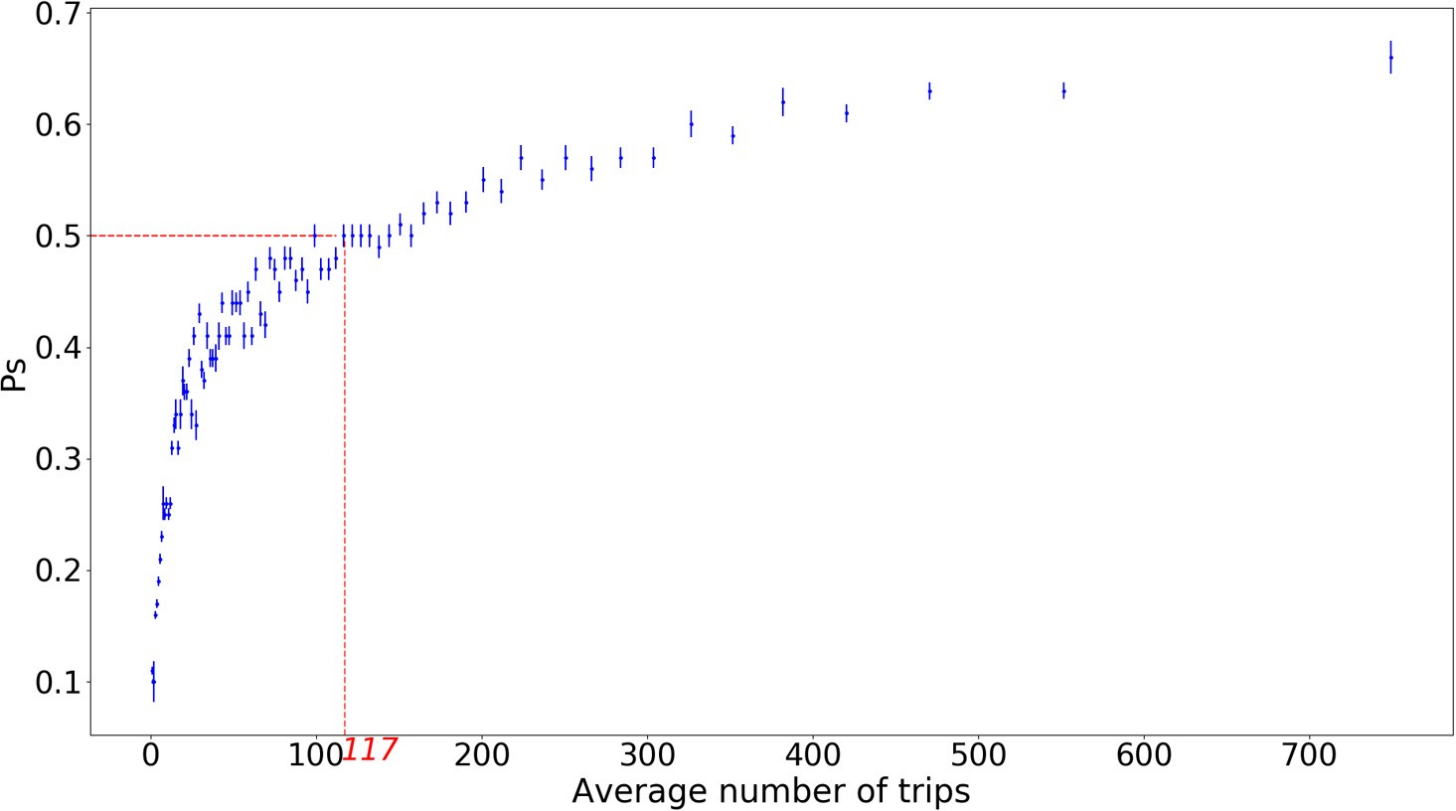
Probability to stay in the system at the end of an adapted year (*P*_*s*_) as a function of the average number of trips during that year. When activity reaches 110-120 trips per year, users are more likely to stay (*P*_*s*_ ≥ 0.5). *P*_*s*_ is computed by ranking person-years by increasing activity and averaging over 2500 person-years.

We noted above that the loyalty of users was correlated to their activity. Fig 3 shows the general trend over all users. It confirms that the higher the intensity of use, the higher the probability *P*_*s*_ to stay in the system. This result can help predicting users’ loyalty.

### 4.2 Long-term users are older, more likely men and more urban than average

In this section, we focus on the most loyal users, i.e. the 25,963 users that have been active for at least 3 years (orange and red rectangles in Fig 2), which we now call ‘long-term’ users. Comparing them to those that leave after a single year reveals interesting facts about their specific characteristics. There exist only small differences in their declared residence area (zip code), but they are older (median age 35 against 24), more likely men (men proportion 62.9% against 49.9%) and live within the Lyon-Villeurbanne urban area (85.3% against 81.7%, all theses differences are highly significant, p-value <2.2*10^−16^). Table 2 shows the proportions of long-term users for different 10-years slices. These statistics were computed on users during their first adapted year. Clearly, loyalty steeply increases with age, from 11.7% for 13-22 years old users up to 52% for 63-72 years old users. Men are over-represented among the long-term users for all ages, but the difference is highly significant among younger users. It would be interesting to understand why there are (comparatively) so few young woman among the most loyal BSS users.

**Table 2:** Description of long-term users characteristics.

Ages	total user number	% men	% long-term users	% men in long-term users
13-22	38,366	52.6	11.7	61.5
23-32	46,696	52.8	17.0	58.8
33-42	17,113	60.0	34.2	65.9
43-52	11,331	54.6	37.6	58.4
53-62	5,572	54.8	45.5	56.4
63-72	1,611	65.8	52.0	66.7

The main point is that these long-term users benefit more from the BSS than the average users, as they use the system more often than them (Fig 2), and remain active for a longer number of years. This original result, which could only be obtained using a long-term dataset, provides further support for the view that BSS are “convenient luxuries” [19] that mostly benefit the already privileged, i.e. male, wealthier and more educated than the average population [13–16, 18, 19, 26, 29].

**Fig 4:**
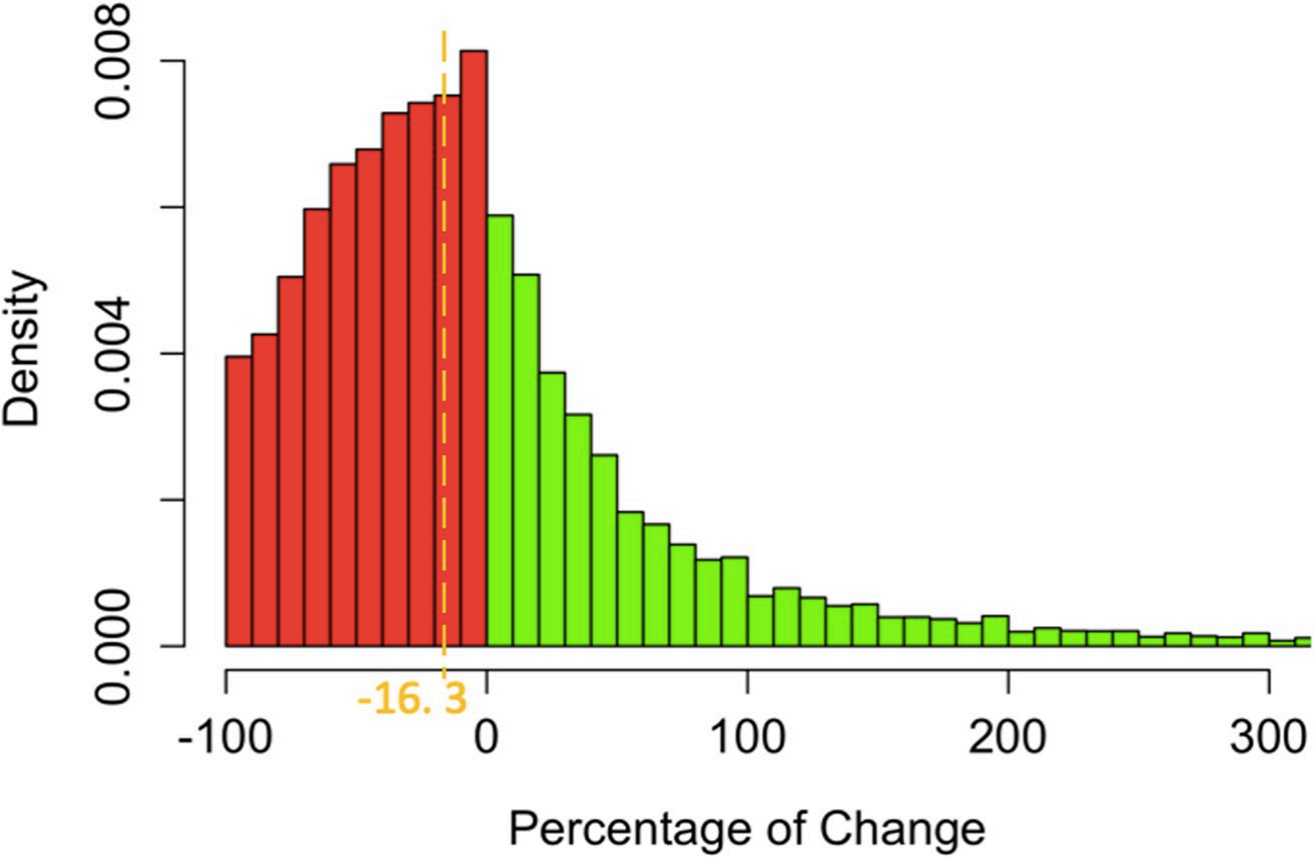
Density distribution of percentage of change in the number of trips per year from one year to another. Percentages are computed for each user that remained active for at least 3 years. The orange dashed line indicates the median decrease of long term users over a year.

Finally, we study how long-term users change their activity over the years. For each user, we computed the percentage of change in the number of trips per year from one year to another. Fig 4 shows that only one quarter (26.5%) maintain their number of trips within a ±20% range. Roughly two-thirds (61.8%) users lower their activity, almost halving it (median decrease 42.3%). The remaining third increases its activity (median increase 42.6%). The median evolution of long term users is a decrease of activity by 16.3%.

## 5 Comparing the 5-years and 1-year classes

We now use our 5-years dataset to test the limitations of classifications based on a single year dataset. We already showed that the activity level observed over a single year is likely to change over time. This relativizes the categorization of a user into a specific user profile based on a single year observation, as in [28]. We now show that the important 1-year category of ‘part-time’ users found in [28], which represents almost a third of all users, actually gathers users that have a *regular* behavior, which appears to be ‘part-time’ because it is observed over a too limited time window.

### 5.1 Computing users classes

To be able to compare the 5-years results with those obtained by [28], we first compute the same 21 normalized features characterizing the activity as in [28]. For each person, these features quantify the intensity and regularity of use over the year (14 features) and the week (7 features). Note that our elementary unit of analysis is the ‘person-year’, i.e. the vector of 21 features for each user and each year. One person can therefore appear several times (up to 5) and change group from year to year. One could adopt a different point of view, using persons as the entities and computing a single vector for each of them, averaged over their whole period of activity. This would have two drawbacks: masking the single user trajectories over the years and comparing vectors computed over different periods (from 1 to 5 years). Comparing the third and fourth columns of Table 3 shows that using the ‘person-year’ or the ‘person’ as the basic entity leads to roughly the same proportions for the different classes.

**Table 3:** Description of user classes found by the k-means for the 21 features.

Class	# person-year	freq	1^st^-year freq	# trips/year
‘one-off’ users	12,164	5.8	5.2	3.0
Week-end cyclist	24,313	11.6	11.8	17
Part-time	7,639	3.6	4.3	80
Regular0	56,849	27.1	27.1	25
Regular1	51,446	24.5	24.9	83
Regular2	29,225	13.9	14.2	175
Regular3	17,183	8.2	8.0	295
Regular4	8,444	4.0	3.6	454
Regular5	2,361	1.1	0.9	695

Note that since the entity is a ‘person-year’, these counts do not directly represent proportions of individuals, because users that stay in the system for long periods are over-represented. However, the comparison with the proportions obtained for year one (third column), which correspond to real users, shows that this effect is relatively weak. *#trips/year* is the median number of trips in a year for each class.

**Fig 5:**
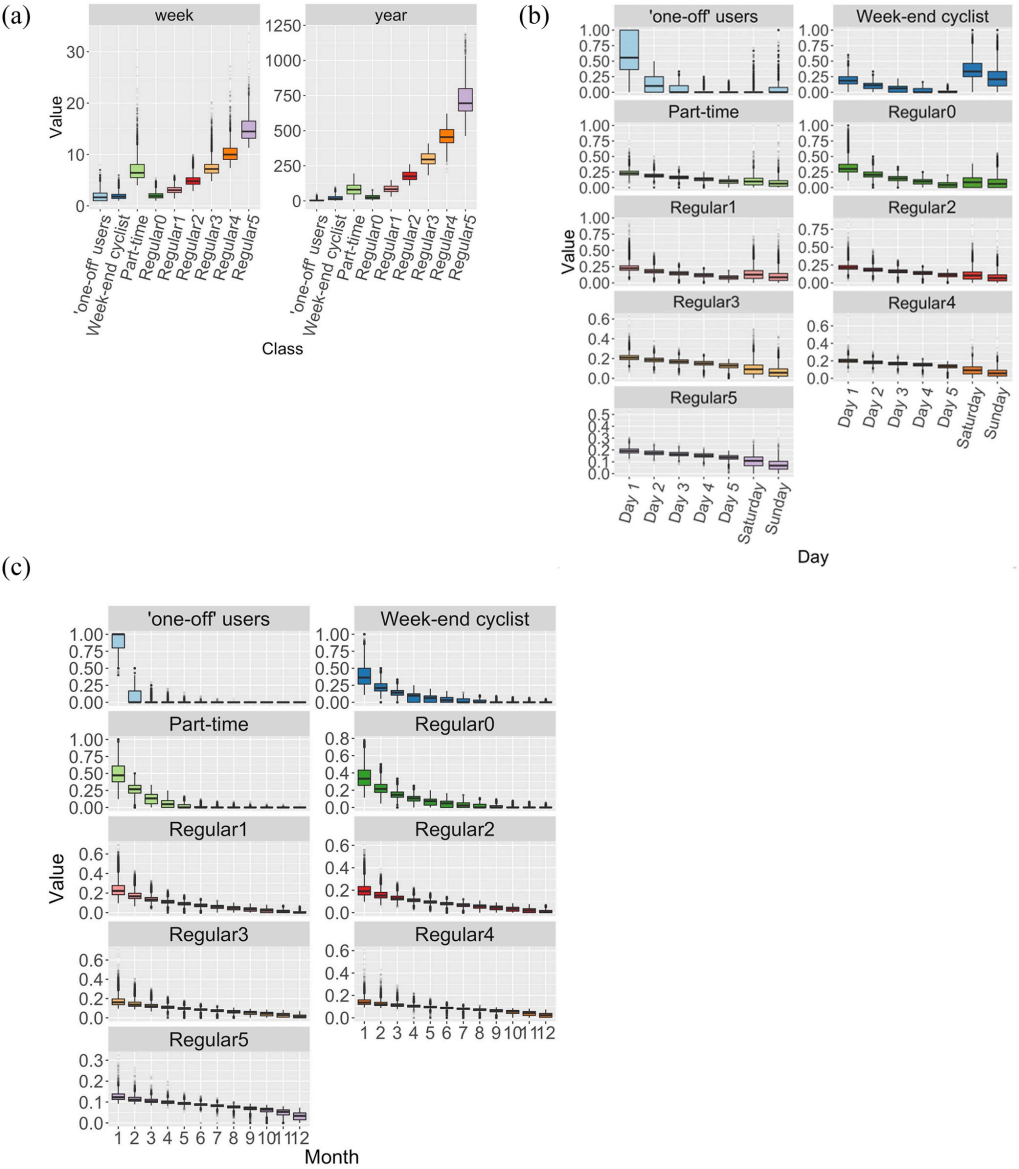
Boxplots of the behavioral patterns at different time scales of the 9 classes. (a) The “value” represents the number of trips per year (right) and per *active* week (left), for each class. A week is considered ‘active’ for a user whenever he/she takes a bicycle at least once. (b) The “value” represents the normalized number of uses for each day of the week and for each class. Week days range from one to five in decreasing order of activity for each user. Saturday and Sunday are computed separately as users’ activity is different on week-ends. (c) The “value” represents the normalized number of uses for each month of the year and for each class.

We then carry out a simple K-means partition in nine clusters, to allow a simple comparison with the previous results. A detailed description of the 9 classes is given in Table 3 and Fig 5.

### 5.2 User classes

The 9 classes correspond to different profiles of use. There are 6% of ‘one-off’ users, who make on average only 3 trips per year, generally the same month and then disappear from the database. Another almost 12% of users are mainly active in week-ends, either for shopping (Saturdays) or leisure (Sundays) (second line of Table 3). The last 6 lines of Table 3 represent users that show a regular activity over the year and differ mainly by their intensity of use, from twice a month (regular0 class, gathering 27% of users) to nearly twice a day (regular5, 1% of users). The part-time class is quite peculiar: We will show below that it can be interpreted as the class where users end up for the last year of activity.

### 5.3 Comparing the one and five years classifications

When comparing the classification obtained here to that found over a single year [28], we note many similarities and a major difference. As in [28], a ‘one-off’ and a ‘week-end’ class are found, with similar proportions, as well as six ‘regular’ classes differing mainly by their intensity of use. The major difference is the ‘part-time’ class, that represents 3.6% of users, instead of 29% for the single year classification (summing their ‘intensive and part-time’ and ‘irregular’ classes). This means that those two 1-year classes mostly gather users that have in fact a *regular* behavior appearing to be ‘part-time’ because they are observed over a too short period of time. For example, a user starting in September 2011 will appear active only for (at most) 4 months, even if she keeps the same activity over the subsequent (unobserved) 8 months.

## 6 Discussion and conclusion

There was so far a lack of analysis of the temporal evolution of *individual* long-term bicycle usage, mainly due to the unavailability of appropriate datasets. So far, there were a few longitudinal analysis of bike sharing systems [26, 30, 31], but these could not investigate *the dynamics of individual users*. Using a unique dataset spanning over 5 years, we have been able to show that:

- There are two main profiles: ‘Leavers’ represent half of the users, quit after a single year and show a low median activity (47 trips); ‘Long-term’ users are more active (median activity of 91 trips in their first year) and remain continuously active for several years (mean time = 2.9 years).
- Long-term users, which benefit most from the BSS, are even more privileged than average users, as male, old and city center residents are over-represented.
- The activity of most (62%) long-term users decreases over time (median variation for all long-term users: -16%).
- 1-year classifications [28] may overestimate part-time users.

Our work suggests further studies on important policy issues which we cannot address for lack of appropriate data. For example, it would be interesting to understand why so many users leave after their first year. There may be personal reasons (moving to other towns…) or reasons related to the BSS (buying personal bikes which are more reliable, switching to scooter or dockless bike use, other motives of dissatisfactions with the BSS service…). It would be interesting to ascertain the relative proportions of each, to help authorities in designing better systems. Finally, more critical assessments are needed about the social benefits of BSS and their real impacts on cities’ sustainability [19, 32, 33].
